# Characterization of the Fecal Microbiota of Pigs before and after Inoculation with *“Brachyspira hampsonii”*

**DOI:** 10.1371/journal.pone.0106399

**Published:** 2014-08-28

**Authors:** Matheus O. Costa, Bonnie Chaban, John C S. Harding, Janet E. Hill

**Affiliations:** 1 Department of Veterinary Microbiology, Western College of Veterinary Medicine, University of Saskatchewan, Saskatoon, Saskatchewan, Canada; 2 Department of Large Animal Clinical Sciences, Western College of Veterinary Medicine, University of Saskatchewan, Saskatoon, Saskatchewan, Canada; The Ohio State University, United States of America

## Abstract

“*Brachyspira hampsonii*” causes disease indistinguishable from swine dysentery, and the structure of the intestinal microbiome likely plays a role in determining susceptibility of individual pigs to infection and development of clinical disease. The objectives of the current study were to determine if the pre-inoculation fecal microbiota differed between inoculated pigs that did (INOC MH) or did not (INOC non-MH) develop mucohaemorrhagic diarrhea following challenge with *“B. hampsonii*”, and to quantify changes in the structure of the microbiome following development of clinical disease. Fecal microbiota profiles were generated based on amplification and sequencing of the *cpn*60 universal target sequence from 89 samples from 18 pigs collected at −8, −5, −3 and 0 days post-inoculation, and at termination. No significant differences in richness, diversity or taxonomic composition distinguished the pre-inoculation microbiomes of INOC MH and INOC non-MH pigs. However, the development of bloody diarrhea in inoculated pigs was associated with perturbation of the microbiota relative to INOC non-MH or sham-inoculated control pigs. Specifically, the fecal microbiota of INOC MH pigs was less dense (fewer total 16S rRNA copies per gram of feces), and had a lower Bacteroidetes:Firmicutes ratio. Further investigation of the potential long-term effects of *Brachyspira* disease on intestinal health and performance is warranted.

## Introduction

*Brachyspira* associated colitis and mucohaemorrhagic diarrhea has re-emerged as a production limiting disease of pigs in North America. In Canada, this re-emergence has been largely associated with a recently described species of *Brachyspira* for which the name “*Brachyspira hampsonii”* has been proposed [1]. The association of *“B. hampsonii*” with disease indistinguishable from swine dysentery caused by *B. hyodysenteriae* has been demonstrated in experimental inoculation studies [2], [3].

The detection of pathogenic species including *“B. hampsonii*” in healthy pigs [4] suggests that exposure alone is insufficient to cause disease, and that extrinsic factors related to the host, pathogen and environment play a significant role in *Brachyspira* pathogenesis. At the individual host level, the development of disease following exposure to *Brachyspira* is influenced by complex interactions between the spirochete and the host intestinal microbiota. Results of numerous early studies following the initial association of *B. hyodysenteriae* with swine dysentery suggested a role for indigenous bacterial populations in *B. hyodysenteriae* infection [5]–[8]. This relationship has been investigated indirectly in subsequent decades through studies of the influence of diet on *Brachyspira* susceptibility (reviewed by Alvarez-Ordonez et al. [9]). However, results are contradictory and the microbiological characteristics that define a “susceptible” pig have not been defined. There is also some evidence that *B. hyodysenteriae* infection can result in perturbation of the intestinal microbiota [10], [11], which could contribute to the long-term effects on health and performance that may occur in pigs following recovery from dysentery [12].

In a recent experimental inoculation study with “*B. hampsonii*” clade II strain 30446 [3], 8/12 inoculated pigs developed mucohaemorrhagic diarrhea while 4/12 *“B. hampsonii*” inoculated pigs and 0/6 sham-inoculated pigs did not. Pre-existing immunity in the unaffected pigs was deemed unlikely since all pigs in the study were obtained from a high health farm with no history of swine dysentery or previous laboratory diagnosis of *Brachyspira*, and no use of metaphylatic feed or water medication in the grow-finisher stage. In the current study, we characterized the fecal microbiota of these pigs both before inoculation and at termination in order to determine if the composition of the pre-inoculation fecal microbiota differed between inoculated pigs that did or did not develop mucohaemorrhagic diarrhea, and to quantify changes in the structure of the microbiome following development of clinical disease. Although no specific microbiome profile was associated with susceptibility to development of clinical disease following inoculation, the development of bloody diarrhea in inoculated pigs was associated with a reduction in total 16S rRNA copies per gram of feces, and a lower Bacteroidetes:Firmicutes ratio relative to sham-inoculated pigs and those that did not develop clinical disease following inoculation.

## Materials and Methods

### Ethics statement

Samples used in this study were collected during a previously published experiment that was designed and conducted in accordance with the Canadian Council for Animal Care and approved by the University of Saskatchewan Committee on Animal Care and Supply (Protocol #20110038).

### Fecal samples

All fecal samples utilized in this study were collected during a previously described experimental inoculation trial (Trial 2. Pure Broth Culture Inoculation [3]). Briefly, pigs were obtained from a high health commercial farm in Saskatchewan with no history of swine dysentery or previous laboratory diagnosis of *Brachyspira*, and no use of metaphylatic feed or water medication in the grow-finisher stage. Farm selection was based on history, and results of screening of 3 and 6 week old pigs for *B. hyodysenteriae, B. pilosicoli* and “*B. hampsonii*”. Eighteen pigs were randomly assigned into two groups, inoculated (INOC, n = 12) and control (CTRL, n = 6). Following an 8-day acclimation period, the INOC group was orally inoculated on each of three consecutive days with *“Brachyspira hampsonii”* clade II strain 30446, while CTRL pigs were sham-inoculated with sterile culture media. Fecal consistency was scored daily as: 0 = formed, normal; 1 = soft, wet cement consistency; 2 = runny or watery; 3 = mucoid diarrhea; or 4 = bloody diarrhea. Pigs were euthanized upon development of mucohaemorrhagic diarrhea (6–12 days) or at 14 days post inoculation (dpi) for INOC pigs that did not develop bloody diarrhea, or 15 dpi for CTRL. Two groups were identified among inoculated pigs: one that developed mucohaemorrhagic diarrhea (INOC MH, n = 8) and another that did not present mucohaemorrhagic diarrhea, although soft feces (maximum fecal score of 1) were occasionally observed in these pigs during the post-inoculation period (INOC non-MH, n = 4). Diarrhea was not observed in any of the CTRL group (0/6). Fecal samples were collected from individual pigs on −8, −5, −3, 0 dpi and on the day of euthanasia (terminal day), and samples were stored at −80**°**C until processing.

### DNA extraction and microbiota analysis

Total DNA was extracted from 200 mg fecal samples using a commercial kit (QIAmp DNA Stool Mini Kit, Qiagen Inc., Toronto, Ontario). For microbiota profiles, *cpn*60 universal target PCR was performed as previously described [13] using multiplex-identifier (MID) tagged primers to facilitate sample pooling prior to sequencing. Amplicon libraries were sequenced in pools of 16 MID tagged libraries on a GS Junior instrument according to the manufacturer’s instructions (Roche, Bradford, Connecticut).

A quantitative PCR assay targeting the 16S rRNA gene was performed on all samples, using the primers from Lee et al. [14] and the protocol of Chaban et al. [15] to estimate total bacterial content.

### Sequence data processing and assembly

Raw sequence data were initially processed using default on-rig procedures (Roche, Branford, Connecticut). The resulting SFF files were de-multiplexed and used as input for the mPUMA pipeline [16] using Trinity [17] for assembly of operational taxonomic units (OTU) and BowTie 2 [18] for read mapping and calculation of OTU abundance. OTU sequences were compared to the cpnDB reference database (cpnDB_nr version 20130321) [19] using a combination of BLAST and the Smith-Waterman algorithm (watered-BLAST) [13] to identify the most similar reference sequences and their taxonomic lineages. OTU sequences of at least 150 bp and with at least 55% nucleotide sequence identity to a reference database sequence were retained for further analysis.

### Statistical analysis

Ecological metrics for richness (Chao 1) and diversity (Shannon) were calculated using Mothur [20]. Prior to calculation of richness and diversity, all libraries were subsampled to 1000 reads to avoid interpretation errors due to different sampling depths [21]. Comparisons of ecological metrics, quantitative PCR values and phylum level abundance data was performed using IBM SPSS version 19.0 (IBM Inc., Chicago, Illinois). Specific tests employed for each comparison are described in the context of the results presented below.

Microbial community profiles generated from *cpn*60 amplicon sequence data were compared using Quantitative Insights into Microbial Ecology (QIIME) v1.8.0 using total read counts for all assembled OTU sequences as input. Beta diversity (ecological distance) was calculated using Bray-Curtis dissimilarity. Average pairwise dissimilarities from 100 bootstrapped datasets at 1000 reads per sample were used to generate UPGMA dendrograms within QIIME.

## Results and Discussion

Environmental and host-associated factors, such as temperature fluctuations, transport, mixing of pigs and other stressful management procedures, diet, and the host gut microbiota are thought to influence the pathogenicity of *Brachyspira* [9]. The indigenous microbiota may exclude pathogens, or limit pathogen growth through competition for resources or production of anti-microbial compounds. In this study, we investigated if fecal microbiota composition differs between pigs that developed mucohaemorrhagic diarrhea following inoculation with *“B. hampsonii”* and those that did not, and if infection leading to disease development disturbed the indigenous microbiota structure. The controlled conditions of an experimental inoculation trial provided an excellent opportunity to investigate these questions since many of the environmental variables that would be encountered in a swine production setting were reduced or eliminated.

Microbiota profiles based on the *cpn*60 universal target sequence were generated for 89 samples from 18 pigs (no sample was available for Pig 683, −8 dpi) resulting in 1,041 to 29,520 sequence reads per sample (median 5,231). Sequence data from the study has been deposited to the NCBI Sequence Read Archive (BioProject PRJNA242423). This sequencing depth provided good coverage of the samples, as indicated by all samples having Good’s coverage values ≥0.91 (average 0.98±0.02). Following raw data assembly and removal of non-target sequences, 1,141 OTU sequences were available for analysis, with 180 OTU detected in at least half of the samples. Comparison of the OTU sequences to cpnDB_nr (a curated database of reference sequences, primarily from bacterial type strains) resulted in the identification of 330 different nearest neighbour reference sequences. Nucleotide sequence identity of OTU sequences to reference sequences ranged from 55–100% (median 77%) (Table S1). Bacteroidetes and Firmicutes were the dominant phyla in the fecal microbiota of all pigs across all days (accounting for 93.2±5.2% of the sequence reads in each microbiome profile), with smaller proportions of Proteobacteria and Actinobacteria; consistent with previous descriptions of the swine fecal microbiota based on either the 16S rRNA or *cpn*60 targets [22], [23].

**Figure 1:**
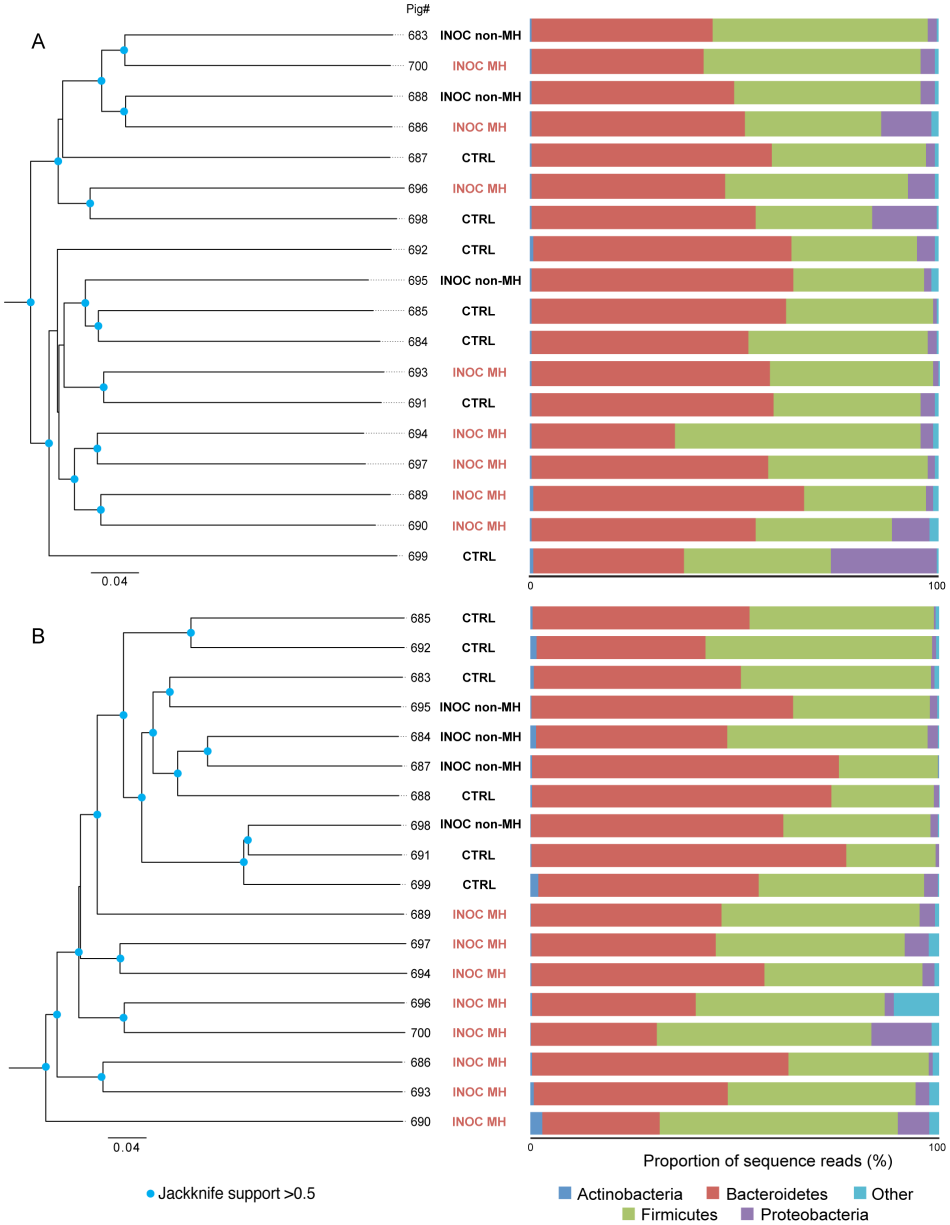
Microbiota profile clustering based on Bray-Curtis dissimilarity calculated from abundance of 1141 OTU sequences (dendrogram, left panel), and proportional abundance of major phyla (stacked bar charts, right panel) in each microbiome. (A) 0 dpi, (B) terminal day.

To determine if pre-inoculation fecal microbiota composition was related to the development of mucohaemorrhagic diarrhea following challenge with “*B. hampsonii*”, richness and diversity, total 16S rRNA content, and *cpn*60 based microbial profiles were examined from −8, −5, −3 and 0 dpi samples from INOC MH and INOC non-MH pigs. CTRL samples were excluded from this analysis since their susceptibility to developing clinical disease upon inoculation with *“B. hampsonii”* was not known. No significant differences in richness (Chao1) or diversity (Shannon index) were observed between groups on any sampling day (Figure S1). The average total bacterial load in pre-inoculation samples estimated by 16S rRNA gene copies was log_10_ 11.2±0.6 per gram of feces. A marginally significant difference in 16S rRNA copies per gram was detected at 0 dpi between non-MH (log_10_ 10.8±0.4 copies/g) and MH pigs (log_10_ 11.3±0.4 copies/g) (Kruskal-Wallis, *P = *0.04) (Figure S2). The significance of this observation is difficult to assess and should not be over-stated since the difference was only detectable on one of the four sampling days and may reflect daily fluctuations in total bacteria numbers. Graphical analysis of the phylum level microbiota profiles illustrated the similarity of composition among pre-inoculation samples (Figure S3), and when proportions of the major phyla were compared among the groups on each sampling day, the only difference detected was that the proportion of Proteobacteria in INOC non-MH pigs was significantly greater than in INOC MH pigs at −8 dpi (Kruskal-Wallis, *P = *0.01) (Figure S4). The difference in proportional abundance of Proteobacteria could be due to factors affecting individual animals prior to arrival at the study site, and it seems unlikely that it was biologically significant since it was not detected on any of the subsequent sample days leading up to inoculation. The overall similarity of pre-inoculation microbiota composition between INOC MH and INOC non-MH groups was further illustrated by the lack of clustering of groups based on Bray-Curtis dissimilarity values for day 0 profiles (Figure 1A).

Inoculation with *“B. hampsonii”* resulted in 8/12 of INOC pigs developing mucohaemorrhagic diarrhea. Terminal restriction fragment length polymorphism analysis has been used previously to detect a “destabilization” of the microbiota of pigs inoculated with *B. hyodysenteriae,* but due to limitations of the technique, the authors lacked the ability to characterize the change in terms of taxa involved [10]. To visualize and quantify any changes to the fecal microbiota of INOC MH pigs in this study, richness and diversity, total 16S rRNA and *cpn*60 microbiota profiles for CTRL, INOC MH and INOC non-MH groups were compared within and between groups at 0 dpi (immediately prior to inoculation) and terminal day (6–12 dpi for INOC MH, 14 dpi for INOC non-MH, and 15 dpi for CTRL).

At termination, total 16S rRNA copies per gram of feces were lower for INOC MH (log_10_ 9.3±0.9 copies/g feces) than either INOC non-MH (log_10_ 10.9±1.0) or CTRL (log_10_ 9.8±1.1), due to the significant drop in total 16S rRNA content between pre-inoculation and terminal day in the INOC MH group (Kruskal-Wallis, *P* = 0.004). This was not unexpected since diarrhea results in increased luminal influx of water, decreasing fecal consistency and increasing the number of bowel movements, leading to dilution and washing out of the luminal microbiota and organisms weakly adhered to the surface of the mucosa. Despite the change in overall density of the microbiota, no significant differences or changes in richness or diversity were detected, suggesting that the decrease in dilution effect was general, affecting the entire population (Figure S2).

Figure 1 shows the relationships of *cpn*60 microbiome profiles of the three groups at 0 dpi and on terminal day based on Bray-Curtis dissimilarity values calculated from abundance of 1141 OTU sequences. Prior to inoculation, the profiles do not cluster according to group (Figure 1A). However, terminal day samples from the INOC MH group cluster separately from the others with good jackknife support (Figure 1B). Further investigation of the proportional abundance of the major phyla in terminal day samples indicated that the INOC MH group had a lower Bacteroidetes:Firmicutes ratio than the CTRL and INOC non-MH together (Paired sample t-test, *P = *0.036) (Table 1). A lower Bacteroidetes:Firmicutes ratio has also been observed in diarrheic relative to non-diarrheic dogs, regardless of the cause of diarrhea [24], suggesting that the environmental changes in the colon brought about by diarrhea may result in an environment more suitable for survival and growth of Firmicutes than for Bacteroidetes. Examination of differences in the abundances of individual OTU sequences between groups did not result in the identification of any significant differences, suggesting that the decrease in Bacteroidetes:Firmicutes ratio was the result of a phylum level effect, rather than a change in abundance of any particular species. Robinson et al. [11] reported a shift in the intestinal epithelial microbiota of dysenteric pigs infected with *B. hyodysenteriae* from Gram positive to Gram negative bacteria, which is not consistent with our observation of a relative increase in Firmicutes (largely Gram positive) in *“B. hampsonii”* affected pigs. However, Robinson et al. used culture-based identification and focused on bacterial populations adherent to the colon epithelium, so direct comparisons with the current study are not appropriate, since the composition of the fecal microbial community is unlikely to be representative of the specialized microbial community closely adhered to the colon epithelium. In humans, the relatively low Bacteroidetes:Firmicutes ratio associated with obesity is thought to contribute to increased risk of *Clostridium difficile* infection in hospital patients with high body mass index [25]. Whether a decreased Bacteroidetes:Firmicutes ratio similarly increases risk of infection with other enteric pathogens in pigs remains to be investigated.

**Table 1:** Bacteroidetes and Firmicutes in the fecal microbiota of inoculated pigs at termination.

	CTRL+INOC non-MH (n = 8)	INOC MH (n = 8)
% Bacteroidetes	59.7±25.6	44.9±11.8
% Firmicutes	37.8±23.3	46.3±7.5
Bacteroidetes:Firmicutes ratio	1.85±0.99^a^	1.03±0.44^b^

a,b Superscript letters within a row indicate significant differences.

*“B. hampsonii”* clade II strain 30446 was detected by species-specific qPCR at levels of log_10_ 6 to log_10_ 8 copies/g feces in INOC MH pigs with bloody diarrhea (fecal score = 4) [3]. However, in the current study we identified an OTU corresponding to *“B. hampsonii”* in the microbiota libraries of only 2/8 of the INOC MH pigs (Pig 690 and 694, which had log_10_ 6.88 and log_10_ 7.81 copies/g *“B. hampsonii”* by qPCR), and it was detected at low abundance in these libraries (1 and 9 reads, respectively). The low rate of detection of *“B. hampsonii”* in the microbiota libraries is perhaps not surprising given the sequencing depth (log_10_ 3–4 reads per sample) and that the levels of *“B. hampsonii”* detected by qPCR in these samples corresponded to on the order of 0.01% of total estimated bacterial population (based on 16S rRNA copy number). It is perhaps a testament to the virulence and adaptation of *“B. hampsonii”* that such dramatic clinical effects can result from the presence of a relatively small pathogen population.

Although we did not identify a particular fecal microbiome profile associated with development of mucohaemorrhagic diarrhea following inoculation with *“B. hampsonii*”, our results demonstrate that pigs that developed severe clinical disease were distinguished from both inoculated pigs that did not develop clinical disease and sham-inoculated controls by a decrease in the Bacteroidetes:Firmicutes ratio of the fecal microbiota. Larger, prospective studies of experimentally or naturally exposed pigs would be beneficial for detecting more subtle changes in the fecal microbiota and the identification of microbiota profiles associated with susceptibility, but these types of studies are currently prohibitive both logistically and financially. The current study was designed to detect differences in the taxonomic composition of the fecal microbiome. Future studies employing metagenomics and metabolomics may reveal important functional differences between the pre- and post-inoculation communities.

In the current study, significant phylum level shifts associated with *“B. hampsonii”* bloody diarrhea were apparent. These initial observations of the effects of an economically significant pathogen on its host lead to hypotheses about the potential long-term effects of these changes in the composition of the microbiota, and their possible contribution to poor performance in pigs recovered from *Brachyspira* disease.

## Supporting Information

Figure S1 **(A) Shannon diversity and (B) Chao 1 estimate of richness for CTRL, INOC non-MH and INOC MH groups on all sample days.**
(TIF)

Figure S2 **Total bacterial 16S rRNA copy numbers per gram of feces in pre- and post-inoculation fecal samples.** Significant differences are indicated with *P* value (Kruskal-Wallis, *P<*0.05).
(TIF)

Figure S3 **Pre-inoculation fecal microbiome profiles for (A) CTRL, (B) INOC non-MH and (C) INOC MH.** Data is shown as proportional abundance of sequence reads assigned to major phyla.
(TIF)

Figure S4 **Proportion of sequence reads identified as Proteobacteria detected in pre-inoculation fecal samples from MH (n = 8) and non-MH (n = 4) pigs.** A significant difference between the two groups was observed at dat −8 p.i. (Kruskal Wallis, *P = *0.01).
(TIF)

Table S1 **OTU table with read counts and nearest neighbours.**
(XLSX)
