# Supplementary figures and images for: Characterization of the Fecal Microbiota of Pigs before and after Inoculation with “Brachyspira hampsonii”
Source: PLoS One. 2014 Aug 28;9(8):e106399. doi: 10.1371/journal.pone.0106399 (PMC4148400; doi:10.1371/journal.pone.0106399)

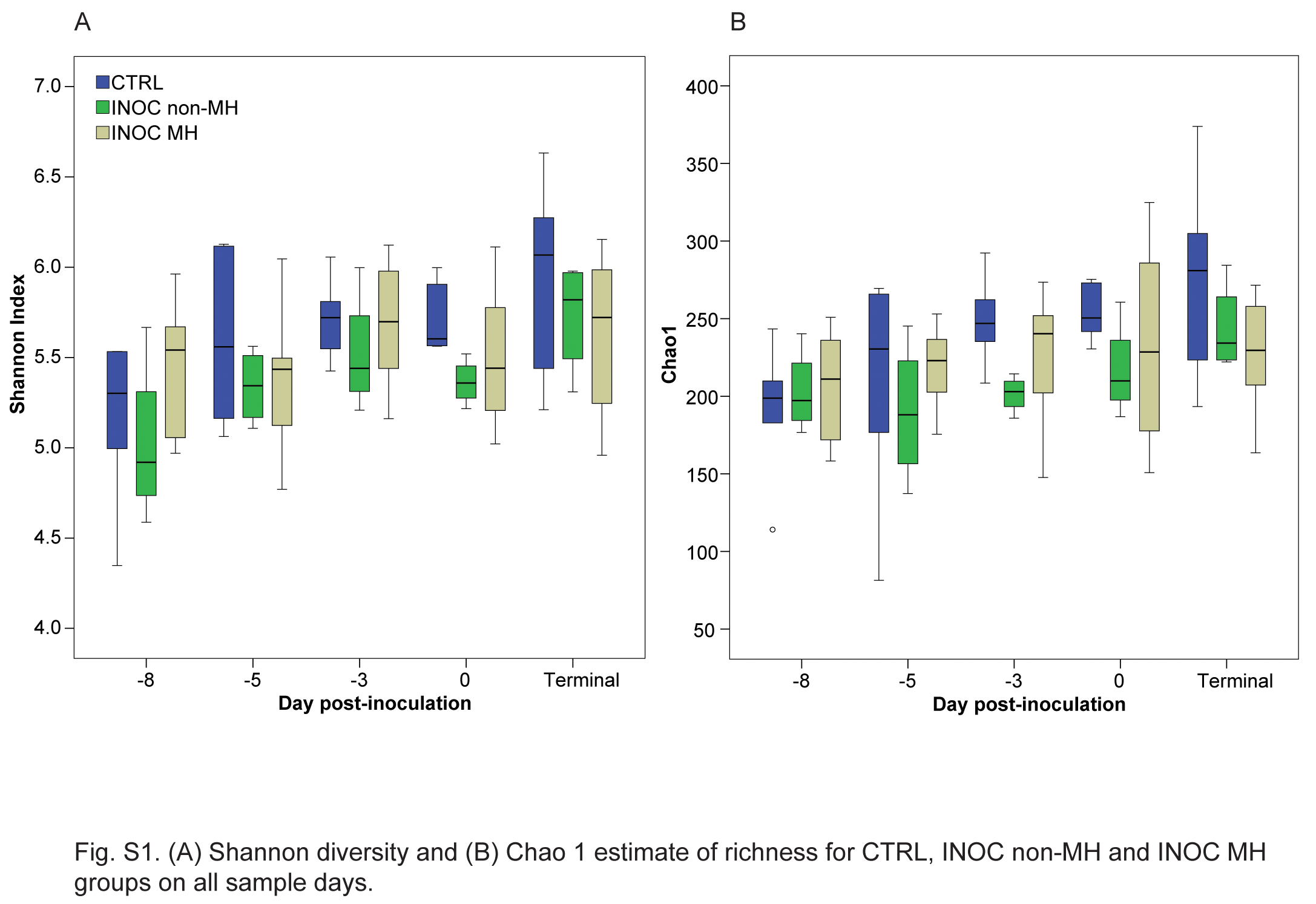

Supplement: Figure S1 — (A) Shannon diversity and (B) Chao 1 estimate of richness for CTRL, INOC non-MH and INOC MH groups on all sample days. (TIF) [file pone.0106399.s001.tif]

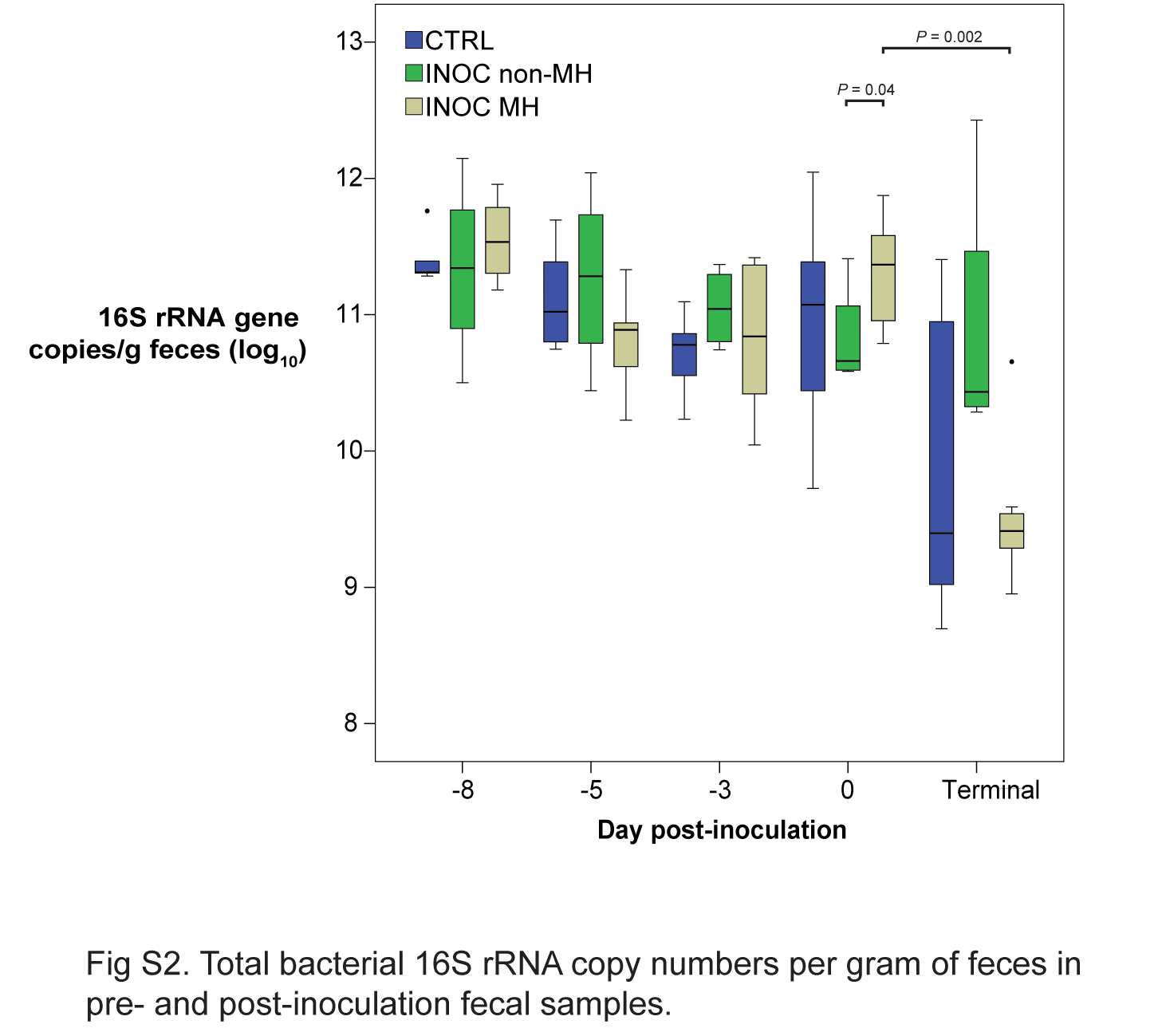

Supplement: Figure S2 — Total bacterial 16S rRNA copy numbers per gram of feces in pre- and post-inoculation fecal samples. Significant differences are indicated with P value (Kruskal-Wallis, P<0.05). (TIF) [file pone.0106399.s002.tif]

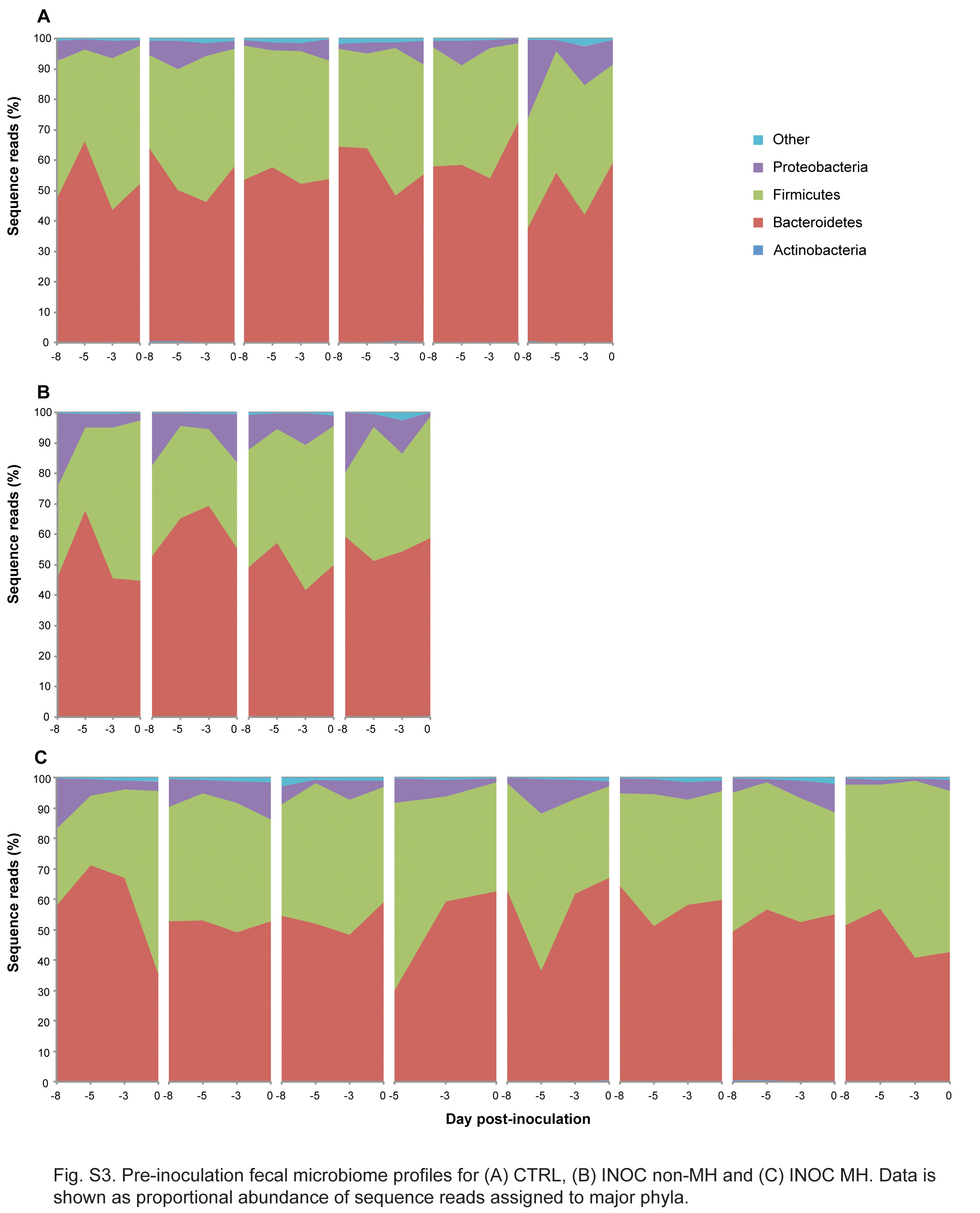

Supplement: Figure S3 — Pre-inoculation fecal microbiome profiles for (A) CTRL, (B) INOC non-MH and (C) INOC MH. Data is shown as proportional abundance of sequence reads assigned to major phyla. (TIF) [file pone.0106399.s003.tif]

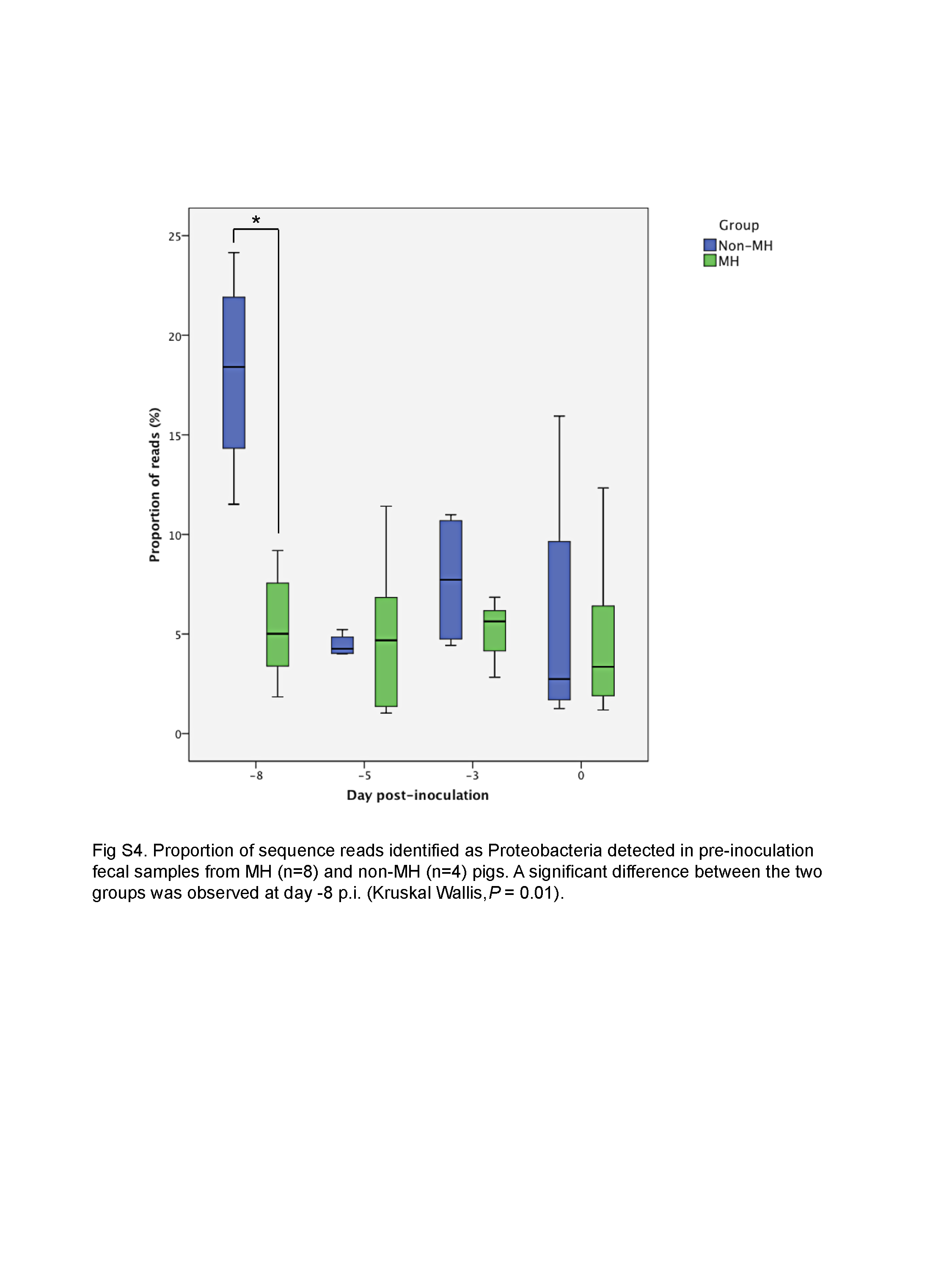

Supplement: Figure S4 — Proportion of sequence reads identified as Proteobacteria detected in pre-inoculation fecal samples from MH (n = 8) and non-MH (n = 4) pigs. A significant difference between the two groups was observed at dat −8 p.i. (Kruskal Wallis, P = 0.01). (TIF) [file pone.0106399.s004.tif]
